# The Impact of Radiotherapy Dose on Local Control of Ewing's Sarcoma of Bone

**DOI:** 10.1080/13577149778452

**Published:** 1997-03

**Authors:** Neil G. Burnet, Judith M. Bliss, Clive L. Harmer

**Affiliations:** 1 Sarcoma Unit Royal Marsden Hospital London UK; 2 Section of Epidemiology Institute of Cancer Research Surrey UK; 3 Department of Clinical Oncology Oncology Centre (Box 193) Addenbrooke's Hospital Hills Road Cambridge CB2 2QQ UK

## Abstract

*Purpose*. Improvements in the systemic management of Ewing's sarcoma of bone over the last 20 years have led to a dramatic improvement in survival. The corollary is that treatment of the primary disease requires re-evaluation, since a significant number of patients still suffer local relapse.

*Patients*. The effect of radiation dose on local control was reviewed in a series of 96 patients treated between 1967 and 1986. Seventy-four had no metastases at presentation (M0), 22 had metastases (M1). The 5-year survival of all patients was 28%, and of M0 patients alone 37%. Although these figures are poor by today's standards, they are consistent with published studies whose patients were enrolled during the same calendar period. Although most deaths occurred by 5 years, survival continued to fall beyond 10 years, which has implications for follow-up in future studies.

*Results*. The local control (LC) rate at 5 years was 56% for all patients and for M0 patients analyzed separately. There was no difference in either LC or survival between the first and second decades of the study. Primary site was a significant determinant of survival and local control, with better outcome for limb tumours compared to pelvic primaries. Chemotherapy also had a major effect on LC. Radiotherapy improved the probability of LC. Omission of radiotherapy, or a dose <40 Gy, was ineffective. In the dose range 40–66 Gy, there was no evidence of a dose–response relationship.


*Sarcoma* (1997) 1, 31–38

ORIGINAL ARTICLE

### The impact of radiotherapy dose on local control of Ewing's sarcoma of bone

NEIL G. BURNET,[1] JUDITH M. BLISS[2] & CLIVE L. HARMER[1]

[1]*Sarcoma Unit, Royal Marsden Hospital, London &* [2]*Section of Epidemiology, Institute of Cancer Research, Surrey, UK*

**Abstract**

*Purpose.* Improvements in the systemic management of Ewing's sarcoma of bone over the last 20 years have led to a dramatic improvement in survival. The corollary is that treatment of the primary disease requires re-evaluation, since a significant number of patients still suffer local relapse.

*Patients.* The effect of radiation dose on local control was reviewed in a series of 96 patients treated between 1967 and 1986. Seventy-four had no metastases at presentation (M0), 22 had metastases (M1). The 5-year survival of all patients was 28%, and of M0 patients alone 37%. Although these figures are poor by today's standards, they are consistent with published studies whose patients were enrolled during the same calendar period. Although most deaths occurred by 5 years, survival continued to fall beyond 10 years, which has implications for follow-up in future studies.

*Results.* The local control (LC) rate at 5 years was 56% for all patients and for M0 patients analyzed separately. There was no difference in either LC or survival between the first and second decades of the study. Primary site was a significant determinant of survival and local control, with better outcome for limb tumours compared to pelvic primaries. Chemotherapy also had a major effect on LC. Radiotherapy improved the probability of LC. Omission of radiotherapy, or a dose $<40$ Gy, was ineffective. In the dose range 40–66 Gy, there was no evidence of a dose–response relationship.

**Key words:** *Ewing's sarcoma, local control, radiotherapy dose–response.*

### Introduction

Ewing's sarcoma is the second commonest tumour of bone in children with an annual UK incidence of approximately 1.7 cases per million, representing 1–2% of childhood tumours,[1] and between 6–15% of all primary bone tumours.[2] It arises almost exclusively in children and young adults. Although not a common tumour by adult standards, its importance lies in the age of population affected and its potential curability.

The role of radiotherapy in the management of Ewing's sarcoma has changed completely since James Ewing first described a 'diffuse endothelioma of bone'.[3] Ewing's sarcoma is essentially a systemic disease requiring systemic treatment. Before chemotherapy became available 20–30 years ago, long-term survival rates were as poor as 10%.[4] Outcome was determined by metastatic disease, with control at the primary site being of less importance. Since the introduction of intensive combination chemotherapy, survival has improved considerably, with some reports of long-term survival being as high as 50–70%.[5,6] As chemotherapy has reduced death from metastatic disease, local control (LC) has once again become more important.

In the management of Ewing's sarcoma, a number of unresolved issues persist in the integration of the treatment modalities, including the roles of radiotherapy and surgery, and the scheduling of radiotherapy and chemotherapy. An additional important question which has attracted less attention is the optimal dose of radiotherapy required to achieve local control without inflicting unacceptable normal tissue damage. Although Ewing himself observed response to radiotherapy,[3] there is comparatively little in the literature on this topic, and all modern studies are complicated by the fact that chemotherapy has a substantial effect on local disease. This study has examined the effect of radiotherapy dose on LC, with the objective of contributing information on dose–response in Ewing's sarcoma.

### Patients and methods

### *Patient details*

All cases of Ewing's sarcoma of bone referred to the Royal Marsden Hospital (RMH) during the period

Correspondence to: N. G. Burnet, Department of Clinical Oncology, Oncology Centre (Box 193), Addenbrooke's Hospital, Hills Road, Cambridge CB2 2QQ, UK. Tel: +44 1223 217074; Fax: +44 1223 217094; E-mail: neilb@argonet.co.uk.

1357-714X/97/010031-08 ©1997 Journals Oxford Ltd

**Table 1.** *Patient and treatment details recorded*

| Details recorded |
|---|
| Age, sex |
| Nature and duration of symptoms at presentation |
| Site of primary tumour |
| Presence or absence of metastases |
| Site or sites of metastases if present |
| Definitive surgical procedure*—resection, amputation |
| Radiotherapy details—dose, fractionation, overall time; timing with respect to chemotherapy and surgery |
| Chemotherapy details—drugs used and duration; timing with respect to radiotherapy and surgery |
| Date of relapse |
| Location of relapse—local, and first distant site |
| Treatment on relapse |
| Date of death or last follow-up |
| Local control or not—at time of death or last follow-up |

*All patients had a diagnostic biopsy.

1967–1986 inclusive were identified using the hospital database and cross-referenced against the Histopathology Department disease register. All histology was reviewed at the RMH and only those cases confirmed as Ewing's sarcoma were retained. Four small round cell tumours of bone without a more specific diagnosis and 10 Ewing's sarcomas arising in soft tissue have been excluded from the analysis.

Information on patients was obtained retrospectively as shown in Table 1. It proved impossible to collect details of tumour size or volume. In the earlier years of the study, CT and magnetic resonance imaging (MRI) were not available, so measurements of the extent of the intramedullary component and soft tissue extension could not be made. For patients treated later, where scanning had been carried out, tumour size had not been recorded. Levels of lactate dehydrogenase (LDH) had not been measured routinely. No attempt was made to detail the chemotherapy doses because it was felt that this information might be inaccurate when collected retrospectively. However, details of drugs used and intended doses, as well as the timing of chemotherapy in relation to radiotherapy and surgery, were collected.

For radiotherapy treatments, details of time, dose and fractionation were collected. All patients were treated with megavoltage photons, except for four patients treated palliatively with low doses ($<24$ Gy, equivalent to 2 Gy/fraction). The margins of radiotherapy fields around the primary tumour could not be extracted retrospectively, partly because tumour extent could not be accurately assessed. A wide range of doses was found, particularly in the first decade of the series. However, the wide variation in radiotherapy dose has allowed an attempt at evaluation of dose–response, which would have otherwise been impossible.

*Radiotherapy dose fractionation*

The variation in dose, fractionation and overall treatment time made direct comparison of dose equivalence difficult and so the nominal standard dose (NSD) method was chosen to estimate the relative effect of different dose-fractionation schedules.[7] This formula uses the relationship

$$\mathrm{NSD} = \mathrm{Dose} \times T^{-0.11} \times N^{-0.24}$$

where NSD is expressed in rets, $T$ is the overall treatment time in days and $N$ is the number of fractions. It was assumed that for every course of radiotherapy including a complete week, a weekend would also have been included, so that a 30-fraction course would have taken 42 days. The formula was used to express treatments as equivalent total doses at 2 Gy/fraction. However, the NSD method incorporates considerable assumptions.[8] The indices in the equation were originally based on the study of acute-reacting 'normal' tissues, rather than tumours. Nevertheless, in at least one study, NSD was found to fit clinical tumour data better than the linear-quadratic model.[9] Although generally preferred, the more modern linear-quadratic model does not, in its simplest form, account for differences in overall treatment time and therefore cannot be applied here to equate treatment schedules.[10]

*Data analysis*

Patients were categorized into those without metastases at presentation (M0) and those with metastases (M1). Patients with metastases at presentation had a much shorter survival; the analysis has therefore focused on those patients who were M0 at presentation.[11] Although M1 patients were analyzed, they have been treated separately. Due to developments in diagnostic methods, especially CT scanning, it is likely that staging was more accurate in the latter part of the study. Thus, it is possible that some patients classified as M0 early in the study would really have been M1. For LC, a minimum period of 3 months was left before recurrence was monitored, to avoid confusion with failure to achieve control. Thus, patients dying within this period were excluded from calculations of LC. Patients were censored if amputation was performed for reasons other than local recurrence, or upon death, since they were no longer 'available' to develop local recurrence.

In comparing the probability of events in different patient groups, the log-rank test was used, with LC and survival probabilities described on a Kaplan–Meier plot. Where several subsets of a parameter have a natural order, for example radiotherapy dose in those patients receiving radiotherapy, a test of trend across groups was applied, rather than a test of heterogeneity. Patients were adjusted for known prognostic variables by stratification before compari-

**Table 2.** *Characteristics of patients and treatment: 96 patients, treated 1967–1986**

| | | |
|---|---|---|
| Age | Range | 1–59 years |
| | Median | 16 years |
| Sex | Male | 53 |
| | Female | 43 |
| Site | Limbs | 50 |
| | Arm 15 | |
| | Leg 35 | |
| | Pelvis | 24 |
| | Rest of body | 22 |
| Metastases at presentation | Yes | 22 |
| | No | 74 |
| Radiotherapy | Given | 83 |
| | Not given | 13 |
| Chemotherapy | Given | 81 |
| | Not given | 15 |
| Definitive surgery** | Resection | 19 |
| | Amputation | 5 |
| | None | 72 |

*Actual numbers are shown.
**All patients had a diagnostic biopsy.

son. Where possible, results have been adjusted for confounding variables, although it is unlikely that the effects of such factors have been completely removed. For example, the choice of treatment and dose, either radiotherapy or chemotherapy, may have have been influenced by expected prognosis. Due to the relatively small size of the cohort, full analysis via multivariate methods was not possible. Hazard ratios are referred to as relative risks throughout.

### Results

Ninety-six patients were available in the study cohort. Patient characteristics and the treatments they received are shown in Table 2, and the distribution of age in Fig. 1. Of the 96 patients, 22 had metastases at the time of presentation (M1). In 11, only one organ was affected; in the other 11, metastatic disease affected two or more systems. Lung was involved in 15 patients, bone in 8, bone marrow in 7, lymph nodes in 2 and liver in 2. No patient had central nervous system disease. Four patients had malaise or fever at presentation, although only two of these had metastases, so these symptoms do not necessarily indicate widespread disease.

The overall 5-year survival of all patients (M0 + M1) was 28% (95% confidence interval (CI) 19–39%), and the 5-year disease-free survival was 14% (95% CI 8–22%). Sixty-eight patients in the study are known to have died, 65 from metastatic disease, two from graft versus host disease following heterologous bone marrow transplant and one from chemotherapy-induced sepsis. All three treatment-related deaths occurred in patients with systemic relapse. Although most deaths occurred by 5 years, a few patients succumbed later; one patient died of disease in the 11th year after treatment. Of the 22 patients with metastases at presentation, 21 are known to have died and the other patient was lost to follow-up with extensive disease.

The LC rates for all patients were 71% (95% CI 60–80%) at 2 years, 65% at 3 years and 56% (95% CI 41–68%) at 5 years. There was no difference in LC or survival according to sex or age at presentation, and no difference in either LC or survival between the first and second decades of the study.

For M0 patients the overall 5-year survival was 37% (95% CI 25–50%), and the 5-year disease-free survival rate 18% (95% CI 10–28%). Not surprisingly, the difference in overall survival between M0 and M1 patients was highly significant ($p < 0.0005$). Of the 74 M0 patients, 47 have died. The survival fell to 22% at 8 years, and 16% at 10 years. It should be noted that the number of patients available 'at risk' was small from 6 years on, but these late deaths have implications for follow-up in future studies. The LC rates for M0 patients were 73% (95% CI 61–83%) at 2 years, 70% at 3 years and 56% (95% CI 41–70%) at 5 years (Fig. 2). Two patients relapsed locally beyond 5 years, at 9 and 10.4 years, respectively, and both developed metastases at the same time.

Eleven patients had symptoms attributable to tumour for over 2 years before presentation (the longest for 3.5 years). There did not appear to be an increased risk of metastases at presentation with longer duration of symptoms, and there was no difference in LC or survival.

Comparing duration of symptoms, there was no difference in survival or LC. Eleven patients had symptoms attributable to tumour for over 2 years before presentation (the longest for $3\frac{1}{2}$ years). There did not appear to be an increased risk of metastases at presentation with longer duration of symptoms.

For all patients, the site of primary tumour had a highly significant effect on overall survival ($p = 0.001$, adjusted for extent of disease at presentation), but not on LC, categorizing primary site as: limbs, axial skeleton or pelvis, in order of reducing survival. For M0 patients, site had a significant effect on survival ($p = 0.04$). The relative risks of local failure for the axial skeleton, limbs and pelvis were 1, 1.6 and 2.2, respectively. These differences were not statistically significant, because of the small number of local failure events (26 in this group), although the magnitude of the effect is consistent with a clinically important difference.

The administration of chemotherapy appeared to have a potent effect on LC, reducing the relative risk of local relapse in M0 patients to 0.53 (95% CI 0.22–1.26%) (Fig. 3). This was not statistically significant ($p = 0.14$), but very few patients did not receive chemotherapy, so the numbers in this comparison are small, and non-receipt of chemotherapy

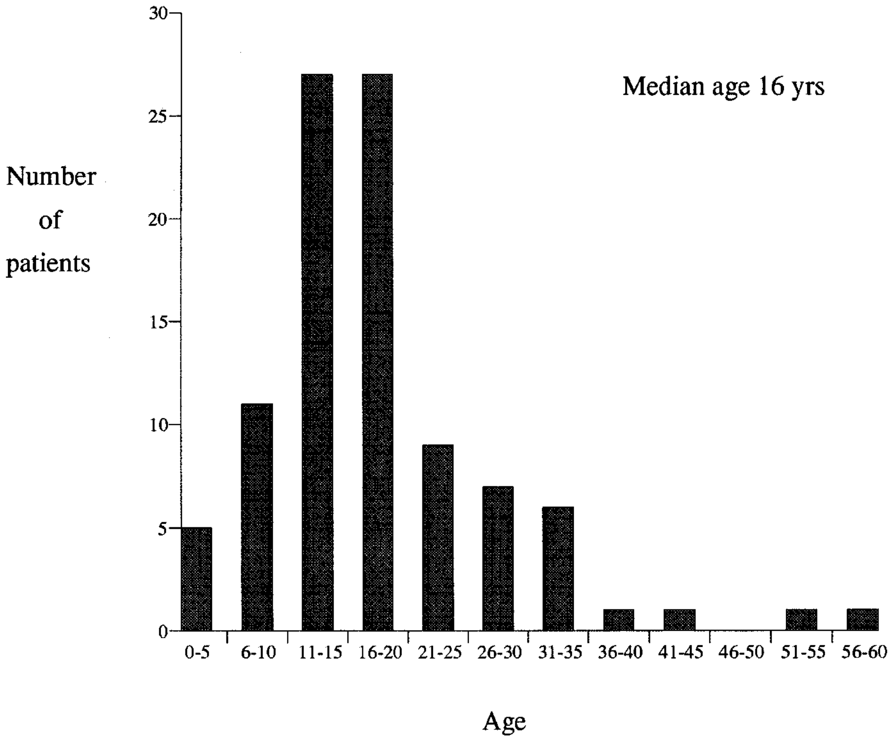

**Fig. 1.** *Age distribution of 96 patients with Ewing's sarcoma of bone.*

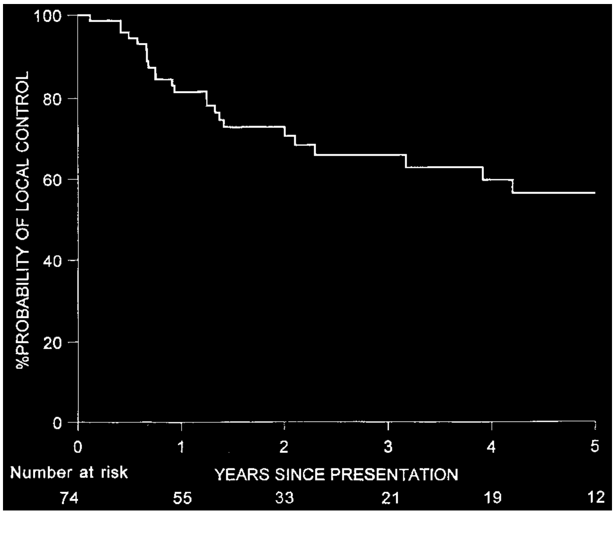

**Fig. 2.** *Kaplan–Meier plot of LC for patients with no metastases at presentation (M0).*

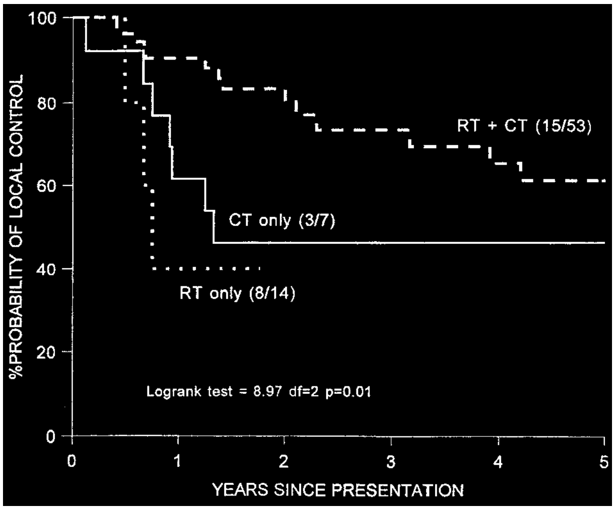

**Fig. 3.** *LC for M0 patients according to treatment modality: radiotherapy (RT) alone, chemotherapy (CT) alone or both. Numbers in brackets refer to* O/N *where* O = *number of events in each group and* N = *number of patients in each group.*

may have been related to widespread disease. In this series, there was no discernible difference in LC or survival with the number of chemotherapy agents administered. High-dose chemotherapy with bone marrow transplantation was not seen to improve LC or survival but this treatment was reserved for patients with very extensive disease at presentation or following relapse. No patients were successfully salvaged after relapse, although one patient survived 5 years.

Surgical resection in M0 patients appeared to improve LC, with the relative risk of local failure falling to 0.74 (adjusted for primary site) following successful removal, but numbers were very small.

The distribution of radiotherapy dose is shown in Fig. 4, with total doses converted to be equivalent to 2 Gy/fraction. Of the 17 patients in the $\geqslant$60 Gy group, 10 received exactly 60 Gy in 30 daily fractions over 6 weeks; the highest dose delivered was 66 Gy. Radiotherapy resulted in an improved proba-

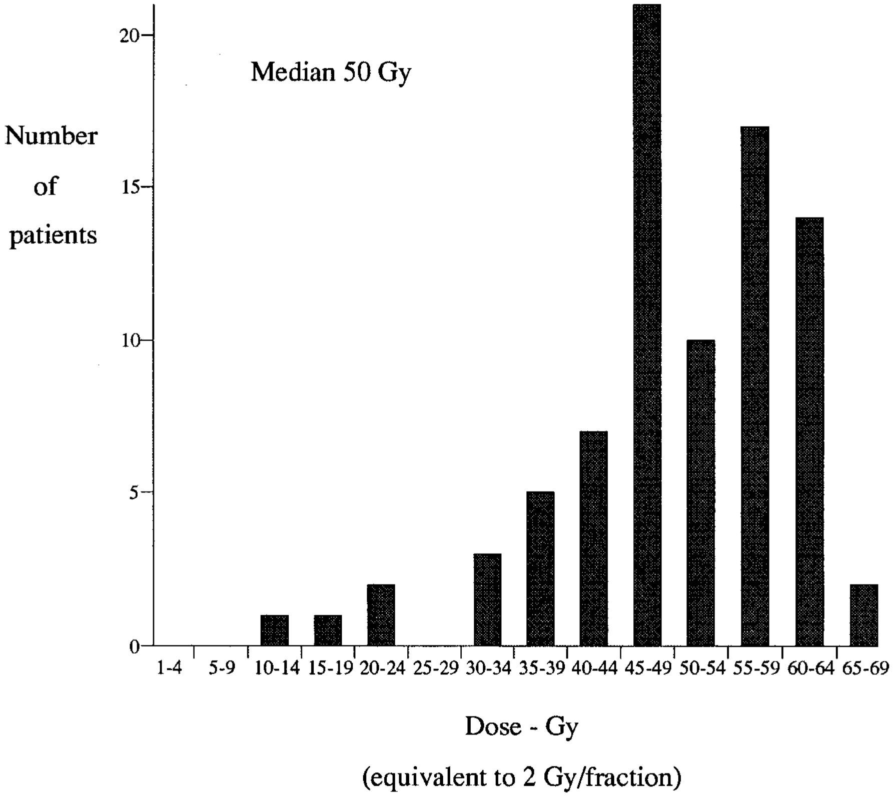

**Fig. 4.** *Dose distribution in the 83 patients who received radiotherapy.*

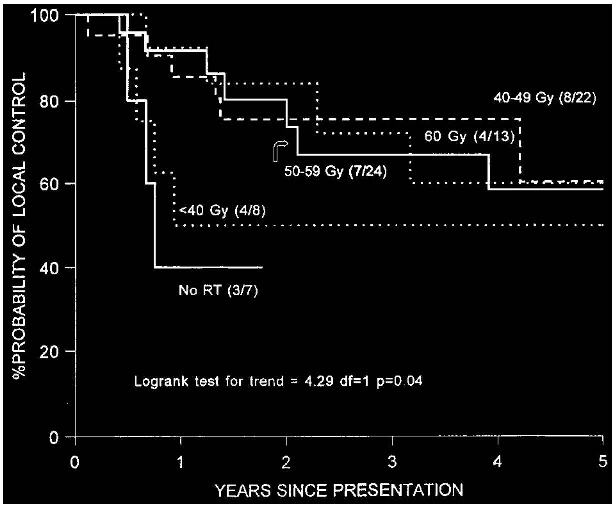

**Fig. 5.** *Local control for M0 patients according to radiotherapy dose, calculated for equivalence to 2 Gy/fraction. Numbers in brackets refer to* O/N *where* O = *number of events in each group and* N = *number of patients in each group.*

**Table 3.** *The effect of radiotherapy dose on LC*

| Radiotherapy dose (Gy) | Relative risk of local failure (95% CI) M0 patients | All patients |
|---|---|---|
| None | 1.0 | 1.0 |
| < 40 | 0.48 (0.1, 2.21) | 0.91 (0.28, 2.89) |
| 40–49 | 0.21 (0.06, 0.87) | 0.29 (0.09, 0.93) |
| 50–59 | 0.21 (0.06, 0.85) | 0.31 (0.10, 0.94) |
| 60–69 | 0.18 (0.09, 0.87) | 0.29 (0.06, 0.85) |
| | $p = 0.04$ | $p = 0.005$ |

Relative risk is shown for patients stratified by primary site. The $p$-values apply to test for trend. See also Fig. 5, which shows the probability of LC with time in M0 patients given different doses.

bility of overall survival when all patients were considered ($p = 0.02$), with an apparent advantage of doses $\geqslant 40$ Gy. Considering M0 patients only, the relative risk of death in those not receiving radiotherapy was 2.5, although this estimate was based on only seven patients and was not significant ($p = 0.15$).

However, this effect on survival is likely to have been caused by omission of radiotherapy from patients with widespread disease at presentation. The same argument applies to the effect of radiotherapy on LC. Omission of radiotherapy, or the delivery of a low dose, greatly reduced the probability of LC. LC rates with different radiotherapy doses for M0 patients are shown in Fig. 5. The log-rank test for trend shows a statistically significant effect for radiotherapy dose ($p = 0.04$), shown in Table 3. A dose of $< 40$ Gy is associated with a considerably reduced chance of LC, and may not even be sufficient for palliation in some cases. All local failures in this group occurred within 1 year of treatment. A comparison of the three groups treated with $\geq 40$ Gy demonstrated no evidence of a dose-response (test for trend $p = 0.82$), although very few local failure events were seen (only 19 in the M0 group).

### Discussion

Progress in the management of Ewing's sarcoma of bone over the last 20 years has led to a dramatic improvement in prognosis, particularly for those patients who are metastasis free at presentation. This has refocused attention on the control of local disease.[12] Ewing originally described the tumour as being 'highly susceptible to radium'.[3] However, a significant local recurrence rate remains. This has led to the increased use of surgical resection, with radiotherapy reserved to follow incomplete surgery, or when surgical resection is impossible. The radiotherapy question which has received most attention in recent years is scheduling with chemotherapy. However, the issues of radiotherapy dose, and the dose–response characteristics of this tumour remain poorly defined.

The purpose of this study was to investigate the effect of radiotherapy dose on local control. There were 96 patients, which at first sight appears a reasonable number. However, the high death rate and losses to follow-up substantially reduced the number of available local failure 'events', particularly when adjusting analysis for possible confounding factors.

### *Problems of retrospective studies*

Drawbacks of retrospective studies include failure to collect data which are now known to be of prognostic value, such as tumour volume, the difficulty of identifying radiotherapy margins around the tumour, and incomplete records of the reasons underlying management decisions. This can lead to analysis being confounded, where the outcome of interest has itself influenced the choice of treatment.

The time period covered by the study saw considerable changes in the methods available for investigating and staging patients, with the introduction of CT and MRI. This did not cause any apparent problems in the analysis but was partly responsible for the increased proportion of M1 patients seen in the second decade of the study (10% rising to 38%), and may have affected management decisions. Dramatic changes also took place in chemotherapy schedules, surgical techniques, and planning and delivery of radiotherapy. All these factors have the effect of increasing heterogeneity in the study group, hampering the interpretation of data. However, provided the danger of over-interpretation is avoided, the advantage of a study of this sort is the collection of information on dose–response, without the use of a two dose-level randomized clinical trial.

### *Study results*

The distribution of age, sex, type and duration of symptoms, and the distribution of primary site in our series were typical (Fig. 1, Table 2).[2] Age had no effect on outcome. This is consistent with other studies, although in some, older age has been associated with a worse prognosis.[12,13]

The site of the primary tumour proved of major prognostic significance, in accordance with other reported experience: limb sites carry a better prognosis than axial skeleton tumours, which in turn are better than pelvic tumours.[12,14–16] It may be that site is really a reflection of the size of tumour at diagnosis rather than being an independent prognostic variable.[17] In the Cooperative Ewing's Sarcoma Study (CESS) 81 trial, a Cox regression analysis identified tumour volume ($<$ or $\geq 100$ cm$^3$) and histological response to initial chemotherapy as the major determinants of prognosis. The primary site was not an independent prognostic factor, probably because of the link to tumour volume.[17]

The presence or absence of metastases at the time of presentation is a major prognostic factor.[12,15,17] This emphasizes the importance of initial staging, and the effect that a change in quality of staging investigations has on comparative results. The poor outlook of M1 patients was the reason for our focusing on metastasis-free patients for the assessment of LC. Chemotherapy had a profound effect on LC (Fig. 3). However, no patients were salvaged after relapse, which is a manifestation of the failure of chemotherapy to sterilize bulky disease.

### *Survival and local control*

The rates of both overall survival and LC in the study were disappointing compared to current studies. It is reasonable to expect patients treated now to have long-term survival rates of around 50%, but such success has been achieved only comparatively recently.[5,11,17] Our results are comparable with other studies reporting on patients treated from the 1960s to the early 1980s.[12,13,18,19] Only the Intergroup Ewing's Sarcoma Study (IESS-I) trial which recruited from 1973 to 1978 reported a substantially higher 5-year survival of 65%, but this was in patients with localized disease at presentation.[5] In IESS-II, recruiting from 1978 to 1982, patients with localized disease excluding pelvic primaries had an overall 5-year survival of around 70%.[20] The CESS 81 and CESS 86 trials have reported 3-year survival rates for patients with localized disease of 55% and 62%, also demonstrating the improvement in survival which has become possible in the last decade.[17]

LC rates have also been rising in more recent studies, reaching 3-year LC rates of around 70–90%.[5,12,17] The local failure rate in CESS 81 was around 50% and is thought to relate partly to poor radiotherapy planning.[6,17] Those studies which cover the early period of chemotherapy, through the 1960s and 1970s, generally report lower rates of LC, in the range of 40–50%, and our results are consistent with these.[16,19]

*Duration of follow-up*

In our study, there was a small rate of attrition due to relapsing Ewing's sarcoma extending out to 11 years, which is the typical experience of studies with long follow-up.[5,12,18] It has occasionally been advocated that disease-free status at 5 years equates to cure, implying that follow-up need not be continued beyond this,[13] but to assess true rates of cure certainly requires longer follow-up. The CESS studies have tended to report 3-year figures, because there are many more 'at risk' patients and most events occur within the first 3 years.[17] There is also a significant incidence of second tumours, partly related to an underlying predisposition in patients with Ewing's sarcoma and partly to treatment, and there is an appreciable incidence of treatment-related complications, some of which are fatal.[13,21] It is therefore mandatory for follow-up to be long enough to record these events.

*Radiotherapy dose–response*

In the pre-chemotherapy era, it was noted that doses of $<40$ Gy resulted in frequent local failure, even though long-term survival was low.[4] More recent studies have failed to demonstrate a dose–response above 40 Gy, although it is generally accepted that higher doses improve LC.[6,17] Our data are entirely consistent with these reports. In the CESS 81 study where patients were randomized to receive either 46 Gy or 60 Gy, no dose–response was found, with local failure just as frequent in the higher dose group. However, LC rates with radiotherapy were poor in this trial until centralized planning was established; this may have confounded any dose effect which might have been present.[6,17] Lack of dose–response has also been seen in other studies.[14,16] In the IESS-I trial, it was felt that this might have been due to the high incidence of death from metastatic disease (almost 50% at 3 years) precluding clinical manifestation of local recurrence.[11]

Some justification for higher doses has come from one study in which LC of bulky tumours ($\geqslant 100$ cm$^3$) was improved by doses of 55–60 Gy.[22] Doses above 60 Gy, in combination with chemotherapy, appear to offer no advantage in LC and have led to impaired functional outcome from normal tissue damage.[23] The consensus is that doses up to about 60 Gy are required for macroscopic disease, although 45 Gy is considered to be adequate for microscopic disease.[6,17]

It is possible that a dose–response does exist but that the search for it has been confounded by small numbers and technical problems.[13,14,17] In addition, a wide variation in intrinsic cellular sensitivity can lead to difficulty in establishing a dose–response. Although *in vitro* data for Ewing's sarcoma are limited, there is a marked spread in sensitivity between tumours.[24]

Assimilating the results from our study and from many others, there is good evidence that doses below 40 Gy are ineffective. In our study, in patients who received no radiotherapy or doses less than 40 Gy, all local failures occurred within 1 year of treatment. This suggests that reasonably high doses are required even for palliative treatment, since patients with metastatic Ewing's sarcoma may survive for many months.

*Conclusions*

In our study, radiotherapy improved the probability of local control. Omission of radiotherapy or a dose of less than 40 Gy proved ineffective for LC, so that low doses may not necessarily be sufficient for palliation. In the dose range 40–66 Gy, there was no evidence to suggest a dose–response.

### Acknowledgements

We would like to thank Miss Jane Regan for obtaining follow-up information and Dr Cyril Fisher for reviewing the pathology. We are also grateful to Professor Ann Barrett, Dr Anna Cassoni and Dr Mark Gaze for stimulating discussions. The patients in this series were referred from many centres, and we are grateful to all those who have furnished updated follow-up information. The Institute of Cancer Research receives financial support from the Cancer Research Campaign.

### References

1 Pinkerton CR, Cushing P, Sepion B. *Childhood cancer management*. London: Chapman & Hall, 1994.
2 Dahun DC, Unni K. *Bone tumours: general aspects and data on 8542 cases*, 4th edn. Illinois: Charles C. Thomas, 1986.
3 Ewing J. Diffuse endothelioma of bone. *Proc NY Pathol Soc* 1921; 21:17–24.
4 Chabora BM, Rosen G, Cham W, *et al.* Radiotherapy of Ewing's sarcoma. *Radiology* 1976; 120:667–71.
5 Nesbit ME, Gehan EA, Burgert EO, *et al.* Multimodal therapy for the management of primary non-metastatic Ewing's sarcoma of bone: a long-term follow up of the First Intergroup Study. *J Clin Oncol* 1990; 8:1664–74.
6 Dunst J, Jürgens H, Sauer R, *et al.* Radiation therapy in Ewing's sarcoma: an update of the CESS 86 trial. *Int J Radiat Oncol Biol Phys* 1995; 32:919–30.
7 Ellis F. Dose, time and fractionation: a clinical hypothesis. *Clin Radiol* 1969; 20:1–7.

8 Bentzen SM, Overgaard J. Time–dose relationships in radiotherapy. In: Steel GG, ed. *Basic clinical radiobiology*. London: Edward Arnold, 1993; 47–54.

9 Hendry JH, Roberts SA. The sensitivity of human tissues to changes in dose fractionation: deductions from the RCR survey among UK radiotherapists. *Clin Oncol* 1991; 3:22–7.

10 Joiner MC. The linear quadratic approach to fractionation. In: Steel GG, ed. *Basic clinical radiobiology*. London: Edward Arnold, 1993; 55–64.

11 Perez CA, Tefft M, Nesbit ME, *et al.* The role of radiation therapy in the management of non-metastatic Ewing's sarcoma of bone. Report of the Intergroup Ewing's Sarcoma Study. *Int J Radiat Oncol Biol Phys* 1981; 7:141–9.

12 Kinsella TJ, Miser J, Waller B, *et al.* Long-term follow-up of Ewing's sarcoma of bone treated with combined modality therapy. *Int J Radiat Oncol Biol Phys* 1991; 20:389–95.

13 Mameghan H, Fisher RJ, O'Gorman-Hughes D, *et al.* J. Ewing's sarcoma: long-term follow up in 49 patients treated from 1967 to 1989. *Int J Radiat Oncol Biol Phys* 1993; 25:431–8.

14 Razek A, Perez CA, Tefft M, *et al.* Intergroup Ewing's sarcoma study: local control related to radiation dose, volume and site of primary lesion in Ewing's sarcoma. *Cancer* 1980; 46:516–21.

15 Glaubiger DL, Makuch R, Schwarz J. Influence of prognostic factors on survival in Ewing's sarcoma. *Natl Cancer Inst Monogr* 1981; 56:285–8.

16 Brown AP, Fixsen JA, Plowman PN. Local control of Ewing's sarcoma: an analysis of 67 patients. *Br J Radiol* 1987; 60:261–8.

17 Dunst J, Sauer R, Burgers JMV, *et al.* Radiation therapy as local treatment in Ewing's sarcoma. Results of the cooperative Ewing's sarcoma studies CESS 81 and CESS 86. *Cancer* 1991; 67:2818–25.

18 Wilkins RM, Pritchard DJ, Burgert EO Jr, *et al.* Ewing's sarcoma of bone: experience with 140 patients. *Cancer* 1986; 58:2552–5.

19 Daugaard S, Sunde LM, Kamby C, *et al.* Ewing's sarcoma. A retrospective study of prognostic factors and treatment results. *Acta Oncol* 1987; 26:281–7.

20 Burgert EO Jr, Nesbit ME, Garnsey LA, *et al.* Multimodal therapy for the management of non-pelvic, localized Ewing's sarcoma of bone: intergroup study IESS-II. *J Clin Oncol* 1990; 8:1514–24.

21 Smith LM, Cox RS, Donaldson SS. Second cancers in long-term survivors of Ewing's sarcoma. *Clin Orthop* 1992; 274:275–81.

22 Barbieri E, Emiliani E, Zini G, *et al.* Combined therapy of localised Ewing's sarcoma of bone: analysis of results in 100 patients. *Int J Radiat Oncol Biol Phys* 1990; 19:1165–70.

23 Tefft M, Lattin PB, Jereb B, *et al.* Acute and late effects on normal tissues following combined chemo and radiotherapy for childhood rhabdomyosarcoma and Ewing's sarcoma. *Cancer* 1976; 37:1201–13.

24 Steel GG, Wheldon TE. The radiation biology of paediatric tumours. In: Plowman PN, Pinkerton CR, eds. *Paediatric oncology—clinical practice and controversies*. London: Chapman & Hall, 1992.
